# Holistic Influence of Multimodal Medical Crowdfunding Affordances on Charitable Crowdfunding Outcome: Systematic Multimodel Analysis Study

**DOI:** 10.2196/75563

**Published:** 2025-09-16

**Authors:** Yuxuan Du, Yujin Yang, Zihe Li, Jiaolong Xue

**Affiliations:** 1 Department of Marketing School of Economics and Management Tsinghua University Beijing China; 2 Department of Marketing Booth School of Business University of Chicago Chicago, IL United States; 3 Graduate School of Economics and Management Tohoku University Sendai Japan; 4 Department of Marketing Business School Sichuan University Chengdu China

**Keywords:** medical crowdfunding, multimodal approach, information affordance, donor behavior, machine learning

## Abstract

**Background:**

Medical crowdfunding has emerged as a critical tool to alleviate the financial burden of health care costs, particularly in regions where economic disparities limit access to medical treatment. Despite its potential, the success rates of medical crowdfunding projects remain low, with only 9% achieving their fundraising goals in China. Previous research has examined isolated factors influencing success, but a holistic understanding of how multimodal affordances—narrativity, visibility, and progress—collectively impact donor behavior and project outcomes is lacking.

**Objective:**

This study aims to investigate how medical crowdfunding affordances, as an integrated system, influence the success of charitable crowdfunding projects. Specifically, it explores the roles of narrativity (textual elements), visibility (visual elements), and progress (dynamic updates) affordances, and how these interact with patient demographics to shape donor engagement and fundraising outcomes.

**Methods:**

A multimodal analysis was conducted using 1261 medical crowdfunding projects from the Shuidichou platform in China. Machine learning techniques (eg, sentiment analysis via SnowNLP) and regression models were used to examine textual content, visual elements, and progress updates. Control variables included patient age, gender, and beneficiary type. Hypotheses were tested using both continuous (success ratio) and binary (success indicator) measures of project success. In total, 6 models were constructed to examine the influences of affordances.

**Results:**

The study found that narrativity affordances—longer titles (model 1a: *P*=.04; model 3a: *P*=.03) and detailed surplus fund descriptions (*P*=.03)—boosted success, while overly lengthy surplus fund explanations had diminishing returns (*P*=.005). Disease mentions in titles increased donations (model 1a: *P*=.01; model 3a: *P*=.003). A neutral tone in the project plan also improved success (*P*<.001). For visibility affordances, a moderate number of progress photos maximized project success, while excessive visuals reduced impact (*P*<.001). Progress affordances followed a similar pattern, with a moderate number of updates enhancing success (*P*<.001). Critically, when all affordances were considered, only progress update frequency retained a strong inverted U-shaped effect on success (*P*<.001). Demographics, particularly age, also influenced donations: patients at both ends of the age spectrum received greater support , while middle-aged individuals received less (model 1b: *P*=.02; model 2b: *P*=.005; model 3b: *P*=.02).

**Conclusions:**

This study advances medical crowdfunding affordance theory by demonstrating the interconnected effects of narrativity, visibility, and progress affordances on project success. Practically, results highlight the importance of strategically crafted titles, targeted demographic disclosures, and balanced progress updates—with moderate update frequency being crucial when controlling all affordances—to enhance donor engagement. Platform designers and project organizers can apply these insights to optimize fundraising outcomes and effectively address health care inequalities. Future research should further investigate visual content analysis and donor psychology to refine engagement strategies.

## Introduction

### Background and Research Aim

By 2030, the economic burden of chronic diseases is projected to reach approximately 343 trillion CNY (US $47 trillion) [1]. In China, the average direct medical expenditure for a case of lung cancer was 39,015 CNY (US $6041) in 2011, with an annual growth rate of 7.55% [2]. Meanwhile, Statista’s 2024 data reported that the average annual disposable income per capita in 2015 was just 21,966 CNY (US $3404)—showing how severe illnesses could push average-income families into poverty.

In light of these financial challenges, crowdfunding has emerged as a key charitable tool, enabling widespread, low-cost support for individuals facing medical expenses without expectation of reciprocation [3]. In China, platforms such as Shuidichou (also called Waterdrop) and Qingsongchou have been developed and supported more than 5 million patients from September 2014 to the end of 2021, raising more than 80 billion CNY (US $12.03 billion) [4]. However, medical crowdfunding projects on these platforms have low success rates: Only 9% of the projects reach their fundraising goals, while 70% achieve just 10% of their target [5]. This underscores the urgent need for research to improve crowdfunding success.

Previous medical crowdfunding research has largely examined success factors in isolation. These factors include cultural proximity [6], social media outreach [7], gender dynamics [8], socioeconomic status [9], linguistic style and structure [10,11], and donor-recipient relationships [3]. More recently, research has begun to explore the properties of information, extending to textual [12-17], visual [12,14,15,18,19], and progress-related [12,13,15,17,20] features of crowdfunding projects.

Most previous studies have focused on GoFundMe [13,14,16,21], which hosts a wide range of projects and primarily serves Western populations, leaving donor behavior in Asian contexts less understood. The majority of Chinese relevant research in China has been conducted using data from Qingsongchou [12,19,22,23] and Tencent Charity platform [15,20]. In contrast, Shuidichou, China’s largest medical crowdfunding platform, is dedicated exclusively to health care projects and remains understudied despite its relevance to Chinese users. While prior research has examined individual informational features, few studies have explored how different cues interact to shape outcomes. To address these gaps, this study applies Gibson’s affordance theory [24], which emphasizes how environments provide actionable information that guides behavior. In the context of medical crowdfunding platforms, the platform serves as the environment, and crowdfunding projects act as information offered to potential donors. Overall, this study aims to address key gaps in prior research [12-17,19,25-29], which has often focused on Western populations and overlooked the interplay among affordances.

Guided by this framework, our study explores: (1) how narrativity affordances (text length, patient demographics, sentiment) influence project success; (2) how visibility affordances (number and type of images) shape donor engagement; (3) how progress affordances (update frequency and sentiment) impact project outcomes; (4) how different types of information—narrativity, visibility, and progress affordances—shape success when controlling for other affordances within the same environment; and 5) how these affordances interact with demographic and contextual factors to shape crowdfunding success.

Building on prior studies, this research responds to Zhang et al [11] by extending the analysis of narrativity affordances beyond project titles to include project descriptions and all textual content. It expands visibility affordances by drawing on Karahanna et al [30] and Zhang et al [31] to examine the number of images across projects. Inspired by Thies et al [32], it investigates progress affordances—particularly update frequency—and incorporates update sentiment, following Zhang et al [3], for a more nuanced content analysis. Additionally, building on findings by Zhou [33], it includes demographic and project characteristics as control variables to assess how affordance effects vary across contexts. By synthesizing insights across modalities, this study constructs a comprehensive multimodal framework encompassing narrativity, visibility, and progress affordances.

### Literature Review

Affordance, as an ecological and psychological concept, has been developed and extended for decades. It was initially proposed by Gibson [24], defining it as the information offered by the environment with references to the abilities of animals to act, mainly in the field of visual perception. Post-Gibson stream researchers [34-36] extend Gibson’s theories and summarize affordance as the property of the environment. Debates and discussions led to further development of Gibson’s theories. Norman [37] was among the earliest to link affordance with human-computer interaction, laying the groundwork for a future extended definition of interaction-oriented affordance. He stresses that users are important because they can perceive and interpret affordances that are built into the design of computers and systems. Later, Jiao et al [38] apply affordances to technologies. He denies and criticizes technological determinism and stresses that affordances are embedded in interactions among users, objects, and technologies. Through perceiving and understanding technologies, users can achieve different goals and outcomes. Heft [34] emphasizes that users can take corresponding actions based on the provided information. Meanwhile, these actions influence information effectiveness.

The rapid rise of the internet and digital technologies has made social media platforms an integral part of daily life, supporting a wide range of activities such as self-promotion, marketing, entertainment, political engagement, and crowdfunding. In recent years, a growing body of academic research has explored the influence of platform affordances on user behavior. For instance, Karahanna et al [30] proposed that individuals use social media to fulfill both psychological and emotional needs, with platform affordances—particularly IT-enabled ones—helping to meet these objectives. Several studies further highlight how platform affordances shape web-based behavior. Zhou [33] finds that changes in social media affordances can causally affect communication among users. Theocharis et al [39] examine how platform features and user interaction reshape political participation. Scharlach and Hallinan [40] argue that IT-enabled affordances transform abstract social values into measurable actions on the web, guiding users’ decisions. In the context of medical crowdfunding, Choy and Schlagwein [41] used the Earthship Kapita case to illustrate how IT affordances on crowdfunding platforms satisfy donors’ psychological needs and motivations, making web-based charity models more effective than traditional offline approaches. Jiao et al [38] deliver a web-based survey and prove that IT affordances effectively influence donors’ perceptions and, therefore, donors’ donation decisions. Collectively, these studies emphasize the central role of affordances in shaping user behavior on the web.

Medical crowdfunding has emerged as a trending method for financing high-cost medical treatments. Severe diseases, exemplified by the far-reaching impact of the COVID-19 pandemic over the past few years, typically put significant economic pressures on patients and their families. Consequently, many resort to medical crowdfunding as a source of economic assistance. To better understand the mechanism of the practice and to optimize the chances of the success of crowdfunding projects, we frame medical crowdfunding as a multimodal information system facilitating the complex interaction between the three central stakeholders: (1) project initiator (organizer or patient), (2) the platform's technology and information infrastructure, and (3) prospective donors. We maintain that when information relevant to the project is properly presented and creates interaction between these actors, the chances of project success can be greatly improved.

Table 1 [12-17,19,20,22,23,42] displays related works and research data of medical crowdfunding platforms over the past several years. Based on the table, the primary studied medical crowdfunding platform includes GoFundMe, Tencent Charity, and Qingsongchou, while the primary methodologies used in related works are text analysis and linear regression. Independent and dependent variables are also acquired as seen in Table 2 [12-17,19,20,22,23,42]. Although various variables are chosen, related works only analyze the impact of the chosen variables on the project success in isolation.

Our study uses data from the Shuidichou platform, which serves a Chinese demographic, to examine both the individual effects of variables and the interaction. In contrast to prior research mentioned that has predominantly relied on data from GoFundMe—a platform primarily used in the United States—our approach expands the demographic scope by focusing on the Chinese context. As the largest crowdfunding platform in China, Shuidichou specializes in health care–related projects, offering a reliable and relevant data source for studying medical crowdfunding. While Qingsongchou is another Chinese platform with comparable functions, it presents a critical limitation: project organizers can alter fundraising goals during a project, potentially introducing bias and compromising data consistency. Therefore, Shuidichou provides a more targeted and stable foundation for our research. In addition to these, while each affordance may individually influence project success, their interactions must also be considered. Beyond studies using field data from platforms like GoFundMe that analyze affordances in isolation, previous experimental research—such as Jiao et al [38]—has used surveys to manipulate specific features and examine their effects. In contrast, our study uses objective field data from Shuidichou and considers affordances simultaneously, capturing how they function together in real-world settings. This integrated approach provides a more realistic and comprehensive understanding of crowdfunding dynamics and offers actionable insights on optimizing information presentation to enhance project effectiveness. In addition, clear explanations on how information presentation can be managed to enhance crowdfunding success are provided, along with examples.

**Table 1:** Medical crowdfunding affordance-related literature in the past 5 years.

Platform	Obs number	Method	Findings	Source
GoFundMe	997	Visual analytics approach	Social media plays a crucial role in boosting medical crowdfunding success.	Ren et al [14]
Qingsongchou	1010	Convolutional neural networks	Emotional-related and credible-related photos increase the donation amount, while rational-related ones decrease it.	Wang et al [19]
Tencent Charity	1891	Linear regression	Project with health-related and disease-related keywords are more likely to succeed	Ba et al [20]
Tencent Le Donation	29,700	Textual analysis	Persuasive language significantly influences donor willingness and trust.	Wu et al [15]
Shuidichou	50,000	Linear regression	Projects with positive expressions achieve higher success rates.	Zheng and Jiang [17]
Qingsongchou	754	Linear regression	Funding goals and project duration can help to predict crowdfunding project performances.	Chen et al [12]
GoFundMe	1830	Univariate, multivariate analysis	The success rate of the project heavily depends on the cancer type and the donor’s social networks.	Holler et al [13]
GoFundMe	92,753	Textual analysis	Hard-to-treat, high-mortality cancers attract more donors. Projects with strong sentiment words and female beneficiaries are more likely to succeed.	Zhang et al [16]
GoFundMe	243,795	Topic model	Images featuring younger individuals, more people, and more smiles significantly improve the success of projects.	Wang et al [42]
Qinsongchou	116,282	Linear regression	Air pollution negatively impacts donations for critical illness, increasing negative emotions.	Hua et al [22]
Qingsongchou	84,712	Linear regression	Key factors such as patient age, disease type, geographical location, and donation target, influence the success of projects.	Zhang et al [23]

**Table 2:** Affordance indicators chosen by the works in Table 1.

Authors	Independent variable				Dependent variable			
	Textual	Visual	Progress	Success rate		Raised amount	Donation number	Willingness
Ren et al [14]	✓	✓		✓				
Wang et al [19]		✓				✓		
Ba et al [20]	✓		✓	✓				
Wu et al [15]	✓	✓	✓					
Zheng and Jiang [17]	✓		✓	✓				✓
Chen et al [12]	✓	✓	✓			✓		
Holler et al [13]	✓		✓			✓	✓	
Zhang et al [16]	✓			✓				
Wang et al [42]	✓	✓		✓				
Hua et al [22]	✓			✓				
Zhang et al [23]	✓			✓		✓		

### Hypothesis Development

**Figure 1:**
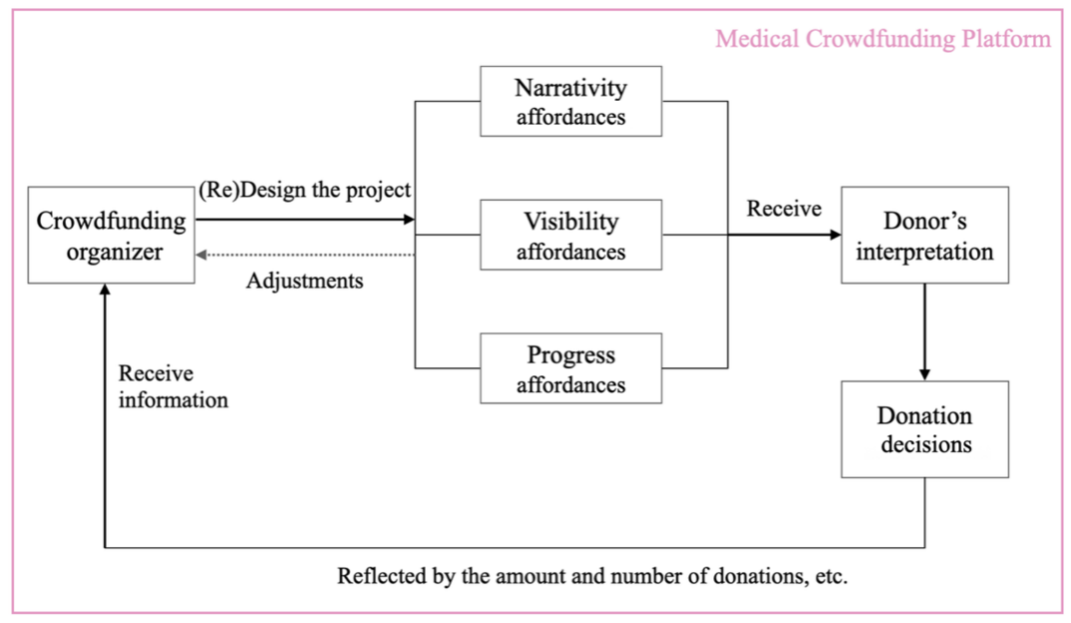
Interaction between project organizers or patients, potential donors, and the platform.

Drawing on affordance theory [24], we conceptualize the crowdfunding platform as an environment in which textual, visual, and progress-related elements function as informational affordances. Similarly, signaling theory states the presence of information asymmetry between sender and receiver [43]. In web-based medical crowdfunding (Figure 1), we have the sender as the crowdfunding project organizer, while the receivers are potential donors online. Senders use affordances to make information signals efficiently to influence receivers, and receivers make informed decisions based on the signals. All interactions among receivers and senders and the information presented on the platform exert a significant influence on donor behavior, ultimately shaping the success rate of crowdfunding projects.

To quantitatively evaluate a project’s success, the most frequently applied metric is the project’s success rate, although its definition is not uniform across earlier studies. For example, Ba et al [20] use a variable termed *Goal_Pro*, where the percentage of raised funds compared with the target donation is used. Likewise, Zhang et al [44] follow the same benchmark. By way of contrast, Zheng and Jiang [17] gauge success by measuring the number of donations received and the donation amount. A lesser-used measure is binary success—whether a project is a success. For example, Parhankangas and Renko [45] create a dummy variable where 1 represents a goal achieved and 0 represents failure. Both continuous and binary measures are used by our study for the dependent variables, providing a richer assessment of project results. Based on these theoretical underpinnings and earlier results (Tables 1 and 2), the following hypotheses are proposed.

**Figure 2:**
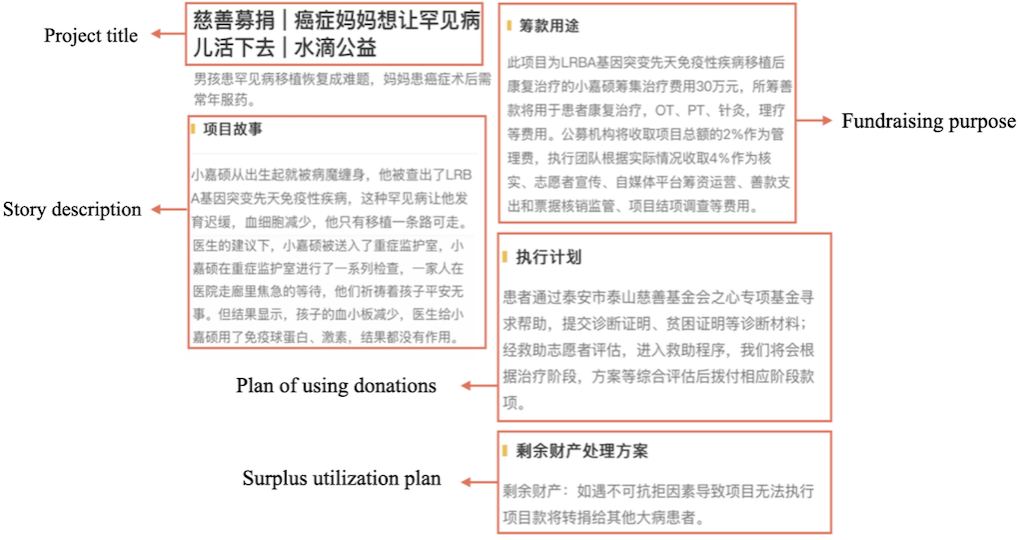
Screenshots of narrativity affordances.

We define narrativity affordance as the action possibilities offered by textual elements written by project organizers. On Shuidichou, these include the project title, project description, fundraising purpose, donation plans, and surplus fund use (Figure 2). To capture donor attention, patients and platform managers must craft descriptions using effective language. Multiple indicators, including text length and sentiment analysis, are selected to evaluate narrativity affordance.

The text lengths of titles and project descriptions have been widely studied, yet findings remain inconsistent. Hou et al [18] and Chen et al [12] claim that a longer text length, especially the length of the story, can attract more attention. However, Zhang et al [44] conclude that there is an inverted U-shaped relationship between story length and the success of a project, meaning that a project with a medium-length description is more likely to succeed. Similarly, Ren et al [14] conclude that the optimal length of a successful project title should be 6 to 11 words. Zhang et al [44] claim that statistically, the length of the title is not significant, and a shorter project description positively influences the success of the project. We develop our first hypothesis as follows.

Text length has an inverted U-shaped relationship with the success of a project.

H1a

Studies also discussed whether project titles or project descriptions should contain disease type, gender, and age in order to improve the success of the project. Chen et al [12], Hou et al [18], and Peng et al [46] all conclude that projects for younger patients, especially children, are more likely to succeed. Zhang et al [44] confirm that if the project title includes disease type, the success rate of the project increases. No past research has directly proved that gender information included in the title and description can impact the success rate of a project. However, Holler et al [13] conclude that female patients are more willing to initiate crowdfunding projects. We develop our second hypothesis as follows.

Disease type, gender, and age in the project title and the project description positively influence the success of a project.

H1b

The sentiments in the title and the project description also influence the success of a project. Researchers apply various methods to capture sentiments. Kim et al [47] designed questionnaires and divided project sentiments into 3 types: responsibility, guilt, and urgency. In the experiment of Wang et al [48], sentiments are measured using scales that include pride and guilt. Although questionnaires and experiments can measure sentiments more precisely, their limitation lies in the need for fully qualified participants; otherwise, accuracy may be compromised. Current techniques can be divided into 2 groups: Lexicon-based and machine learning methods. Agarwal et al [49] construct a domain-specific ontology with ConceptNet and WordNet, extract wordings with natural language processing (NLP), and convert them into a contextual polarity lexicon, classifying sentiments into positive, negative, and neutral ones. Similar lexicon-based methods are used by multiple research works [50-53]. Machine learning techniques are commonly used by extracting and crawling keywords, followed by classification. With such techniques, it is possible to analyze a relatively larger volume of texts and quantify the sentiments in them [54-56].

Previous studies report various findings on sentiments in titles and project descriptions. Zhang et al [57] conclude that the negative sentiments in project titles decrease the success rate, but if these words are in project descriptions, the success rate increases. Hou et al [18] and Zheng and Jiang [17] confirm that if the project description narratives are more positive, the project tends to succeed. We propose a hypothesis as follows.

Positive or negative sentiments of the project title and project descriptions significantly influence the success of a project.

H1c

**Figure 3:**
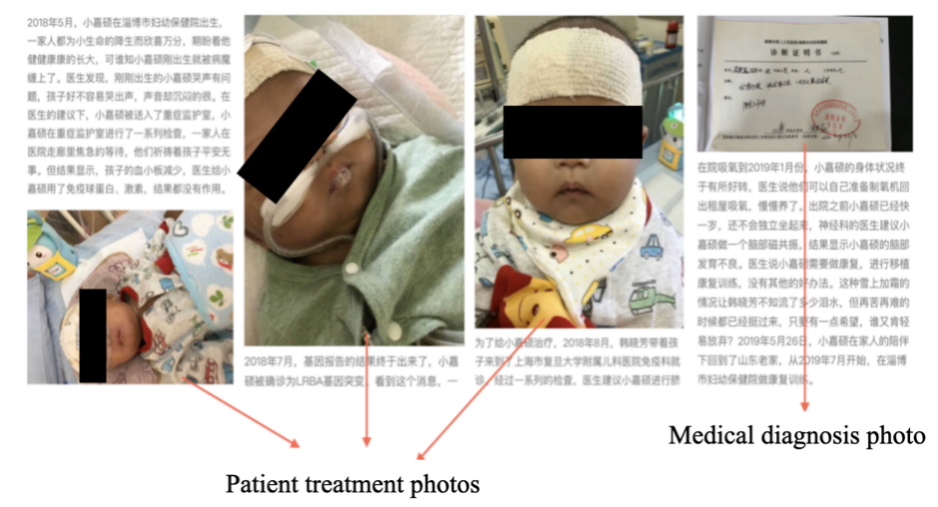
Screenshots of visibility affordances.

We define visibility affordance (Figure 3) as the action possibilities through design and graphical elements, specifically referring to the number of photos in different sections (main page and progress updates). Studies have proven that photos and videos on social media are effective tools for establishing web-based identification and attracting the attention of online users [58,59]. Similarly, posting photos for medical crowdfunding projects can provide potential donors with more direct information, and photo types, such as the current status of patients and ID verification, can influence donor motivation and donor trust [14]. Wang et al [19] list the influence of different types of photos on the success of a project. They point out that photos related to the current health status and treatment status positively influence the success of a project, while those presenting treatment expenses negatively influence the success of a project. Thies et al [32] and Wu et al [15] conclude that the number of photos included positively influences the success of a project. Therefore, our hypothesis is as follows.

The number of photos included in a project positively influences the success of a project.

H2

**Figure 4:**
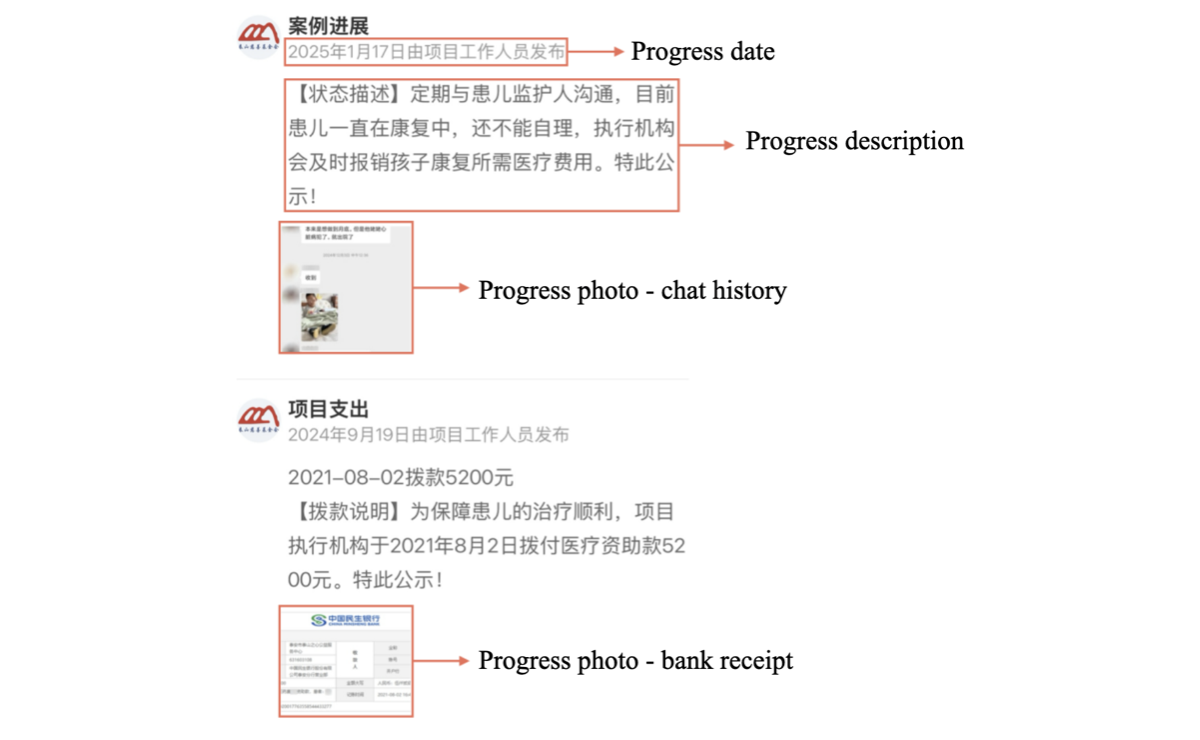
Screenshots of progress affordances.

We define progress visibility (Figure 4) as the action possibilities offered by progress updates, including elements such as patient treatments, donation cash flows, etc. The progress updates of a project can be crucial for the following reasons. First, regularly posting updates establishes the credibility of the project organizer [15], because this shows that the project is transparent and that donors can track their donations. Second, platform managers can structure textual descriptions and use words that convey positive or negative sentiments to evoke donor empathy. Some prior academic research has already highlighted the impact of progress updates. Parhankangas and Renko [45] claim that a higher frequency of progress updates improves donor engagement, thereby enhancing the project’s success. Ren et al [14] and Wu and Peng [60] have similar conclusions. Zhang et al [44] conclude that a high frequency of progress updates with negative sentiment words can significantly increase the success rate of a project. Wu and Peng [60] propose that sentiments in progress updates significantly influence donation amounts. Previous studies also suggest an inverted U-shaped relationship between progress update frequencies and project success. Thies et al [32] and Wu et al [15] mention that when the frequency is too high, donors can be negatively influenced by information overload. We develop 2 hypotheses as follows.

The frequency of progress updates has an inverted U-shaped relationship with the success of a project.

H3a

Positive or negative sentiments in progress updates significantly influence the success of a project.

H3b

## Methods

### Ethical Considerations

This study did not require ethics board review because it did not involve direct interaction with human participants nor the collection of any private or identifiable information. All analyses were conducted on aggregated, anonymized data from public sources. This rationale is consistent with prevailing ethical research standards and common institutional practices for studies of this nature.

### Overview

All data were extracted from disease-related crowdfunding projects on the Shuidichou platform. This study did not involve any surveys or experimental interventions; thus, no RCT registration number is applicable. Launched in July 2016, Shuidichou is China’s leading platform for medical crowdfunding. By the end of 2020, the Shuidichou platform had facilitated over 37 billion RMB (approximately US $5.1 billion) in donations from more than 340 million contributors across 1.7 million medical aid projects, according to its publicly reported data.

According to company profile summaries on Tracxn.com, while GoFundMe remains the leading crowdfunding platform [31], Shuidichou specializes in health care, making it China’s top platform and the world’s third-largest. Backed by US $251 million from major investors like Tencent and IDG Capital, its scale and user base offer a strong basis for analyzing information affordances in crowdfunding success.

On Shuidichou, project pages follow a standardized format. Information—such as the project title, target amount, funds raised, timeline, beneficiary, initiating organization, project leader, and donation recipient—is shown on the right side. On the left, donors can navigate three sections: (1) Project Details, which outlines the patient’s story, fundraising purpose, fund allocation, execution plan, and surplus handling; (2) Project Progress, which provides updates on the patient’s condition and fund use; and (3) Donation Status, which tracks contributions from donors.

To ensure representative results, this study uses a sample of 1261 crowdfunding projects initiated between May 11, 2018, and September 5, 2024. Due to platform restrictions requiring additional authorization for long-expired projects, we extracted data from all publicly available projects as of November 11, 2024, without applying additional selection criteria. As such, we consider the dataset broadly representative of various disease types, while acknowledging that its demographic scope is limited to the Chinese population and that some projects may have been overlooked due to limitations in platform visibility. Among these projects, 1228 out of 1261 (97.38%) have already closed. Given that this study aims to understand how the platform's information management capabilities influence whether a project reaches its funding goal, and recognizing that projects may meet their objectives before the intended end dates, we chose not to exclude any projects based on their completion status.

### Data Extraction

The Shuidichou platform restricts the direct extraction of comprehensive project data. To address this, research assistants manually collected project links to include all available projects for individuals with various diseases. Software engineers then used Python to scrape donor-visible data. Since patient gender, age, and name are not explicitly displayed in a dedicated section, these variables were manually coded.

After removing duplicates, we conducted a comprehensive textual analysis to examine how affordances influence donation behavior across project titles, project descriptions, progress updates, fund purposes, fund use plans, and surplus fund use. All text elements were evaluated for length and sentiment using SnowNLP. Titles and project descriptions were the primary focus, as they conveyed crucial information to donors. We measured text length excluding spaces and punctuation to assess information density and generated dummy variables for age, gender, and disease presence using Python-based text-matching methods.

For sentiment analysis, we preprocessed text using the Jieba Chinese segmentation tool and generated sentiment scores with SnowNLP, which assigns values from 0 (negative) to 1 (positive). SnowNLP is a widely used tool for Chinese sentiment analysis, particularly effective for processing user-generated content such as social media and crowdfunding texts. Prior studies have successfully applied SnowNLP for sentiment analysis in Chinese platforms, demonstrating its suitability for this context [61]. We further analyzed the frequency of positive, negative, and degree words using the Hownet Chinese dictionary [62].

### Variables and Models

#### Dependent Variables

The success of crowdfunding projects is measured in two ways: (1) success ratio: the ratio of the donations received to the funding goal. (2) success indicator: a binary indicator of whether the project met or exceeded its goal. Only 39 projects have fully reached their funding targets. Given the limited number of successful cases, we conducted bootstrap robustness tests for each model using *success_ind* as the dependent variable. Only models that passed the robustness test—indicating low bias and stable results—were considered.

#### Independent Variables

The main independent variable in this study is information affordance, comprising three components: (1) narrativity affordance, which examines how textual elements influence donation behavior through factors such as text length, word frequency, and sentiment scores; (2) visibility affordance, which explores the role of visual information, particularly the number of images in project descriptions and progress updates, in shaping donor engagement; and (3) progress affordance, which analyzes how the frequency and content of patient progress update impact donation patterns over time.

#### Control Variables

The control variables in this study are patient age, patient gender, and an indicator of whether the beneficiary is an individual or a group of recipients.

The multivariable models assess how narrativity, visibility, and progress affordances influence crowdfunding success. Each model is tested with both the success indicator and success ratio using regression analysis and U-tests. Given that each affordance includes distinct elements—such as textual length, which may be reflected in title length, story length, or other components—that may influence donor decisions differently, we analyze them separately to test for differential effects. Models 1-5 assess the individual impact of specific affordance elements on the success ratio, while model 6 takes a holistic approach to examine how narrativity, visibility, and progress affordances collectively influence success.

Models 1 to 3 focus on narrativity affordance, leveraging the textual richness of titles and project descriptions. Model 1 examines titles, model 2 analyzes project stories, and model 3 combines both, along with additional elements such as purpose statements, plans, and surplus fund use, to assess the impact of narrative elements on donation behavior. Model 4 captures visibility affordance by measuring the effect of image counts in project descriptions and progress updates. Model 5 addresses progress affordance by incorporating patient progress updates’ frequency, length, and sentiment to explore how updates shape donation behavior.

Model 6 integrates the 3 affordances into a comprehensive framework. To assess the effect of each affordance type while controlling for the others, we aggregated related variables within each category. Narrative length is calculated by summing all relevant text fields. Demographic information—patient age, gender, and disease type—is combined into a composite index, with higher values indicating more demographic content. Wording valence is derived by subtracting total negative word counts from total positive word counts. Additionally, degree words and their intensity scores from both the title and project descriptions are summed to capture overall linguistic intensity.

## Results

### Descriptive Statistics

#### Overview

Only 39 out of 1261 projects (3%) successfully reached their funding goals. On average, the projects achieved only 18% of their target. To validate our success measures, we examined their correlations with key outcomes. Both the number of donations (*R*=0.5512 and 0.2359) and the amount received (*R*=0.5455 and 0.2167) show strong, significant correlations with success ratio and success indicator (all *P*<.001). Descriptive statistics are provided in Tables S2 and S3 in Multimedia Appendix 1.

#### Robustness Test

To ensure the statistical validity of our results, we conducted robustness tests for both the success ratio and success indicator models. Given the small number of fully successful projects, these tests help confirm that our findings are stable and not driven by random variation. Specifically, we used nonparametric bootstrapping with 1000 replications for models 1 to 6 using both dependent variables.

For the success ratio models, bootstrap analyses demonstrated generally stable coefficient estimates across models 1 to 6, with low bias (< ±0.05) and moderate standard errors (<0.10). However, in model 2, the sentiment score calculated using the SnowNLP algorithm exhibited substantially inflated standard errors (up to 30) and high bias, indicating instability for this predictor. Similarly, in model 3, the indicator for whether the project beneficiary is an individual showed large standard errors (up to 1464.41) and considerable bias, likely due to model complexity and multicollinearity among narrative-related features. These unstable predictors were excluded from interpretation, while the remaining estimates across models 1 to 6 demonstrate solid robustness in explaining the success ratio.

In the success indicator models, bootstrapping with 1000 resamples for models 1 to 3 (focused on narrative affordances) revealed substantial statistical instability. Several predictors showed significant bias (eg, exceeding ±1.0 in model 1 and reaching up to ±1.78×10^13^ in model 3) and substantial standard errors (up to 4.65×10^14^). Consequently, estimates from these models are excluded from further interpretation. In contrast, bootstrap analyses for models 4 to 6, which examine visual, progress, and combined affordances, produced substantially more stable results. Most key predictors in these models exhibited minimal bias (< ±0.05) and moderate standard errors (<0.10), supporting the reliability of these estimates. Therefore, models 4 to 6 are retained for interpretation, given their demonstrated stability.

Collectively, these analyses demonstrate that the majority of models in this study produce stable and reliable estimates, with specific unstable predictors identified and excluded from interpretation. To further assess the robustness of the success ratio models, we conducted a sensitivity analysis by re-estimating the models after excluding projects that fully met or exceeded their fundraising goals. This approach tested whether these extreme cases disproportionately influenced the results. The findings remained consistent, with similar coefficient magnitudes and significance levels, and only minor fluctuations were observed. All results based on the filtered dataset are reported for transparency (Tables S4-S6 in Multimedia Appendix 1). Together, the bootstrap and sensitivity analyses provide a comprehensive assessment of model reliability, while acknowledged limitations ensure cautious, evidence-based interpretation.

#### Regression Results

**Figure 5:**
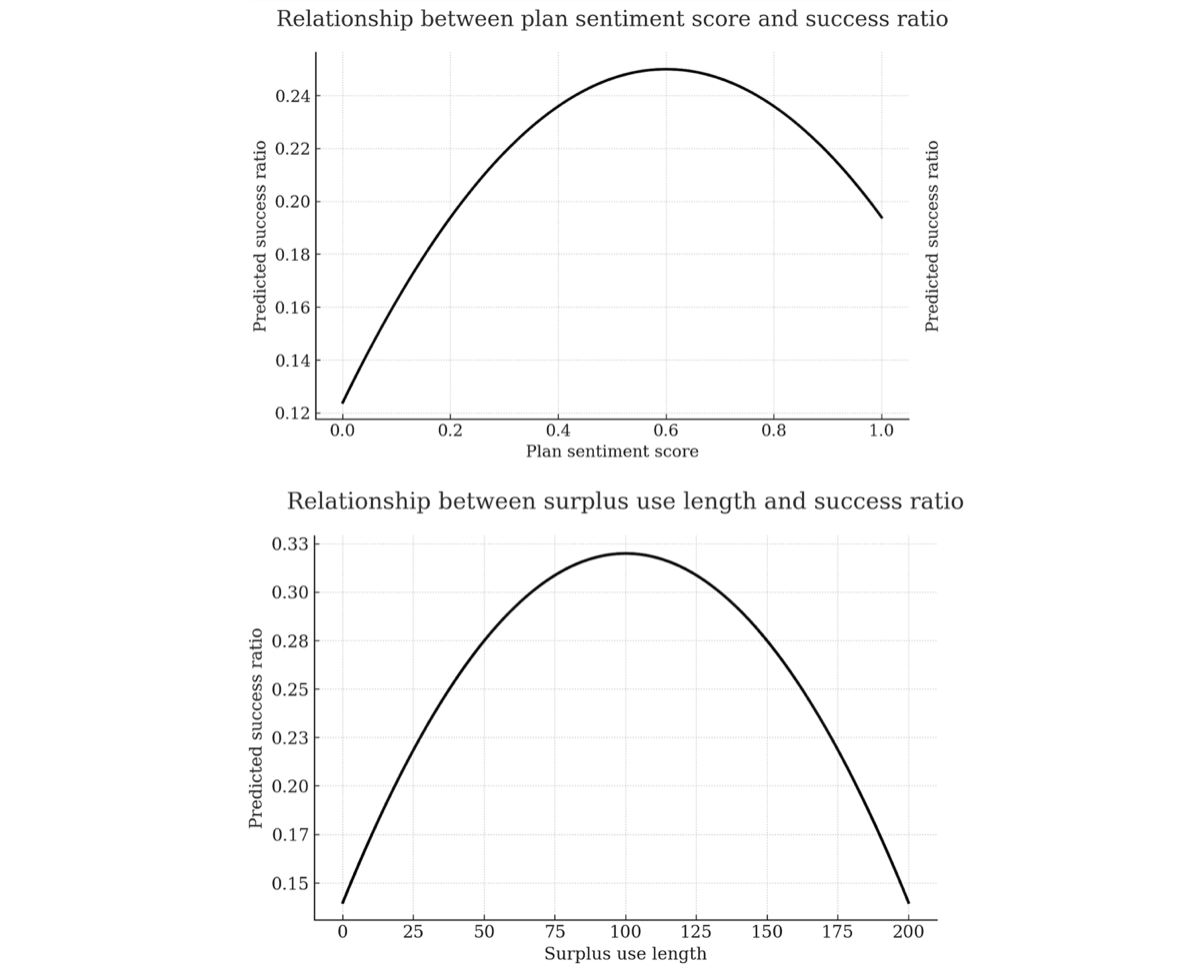
U-test results for model 3.

**Figure 6:**
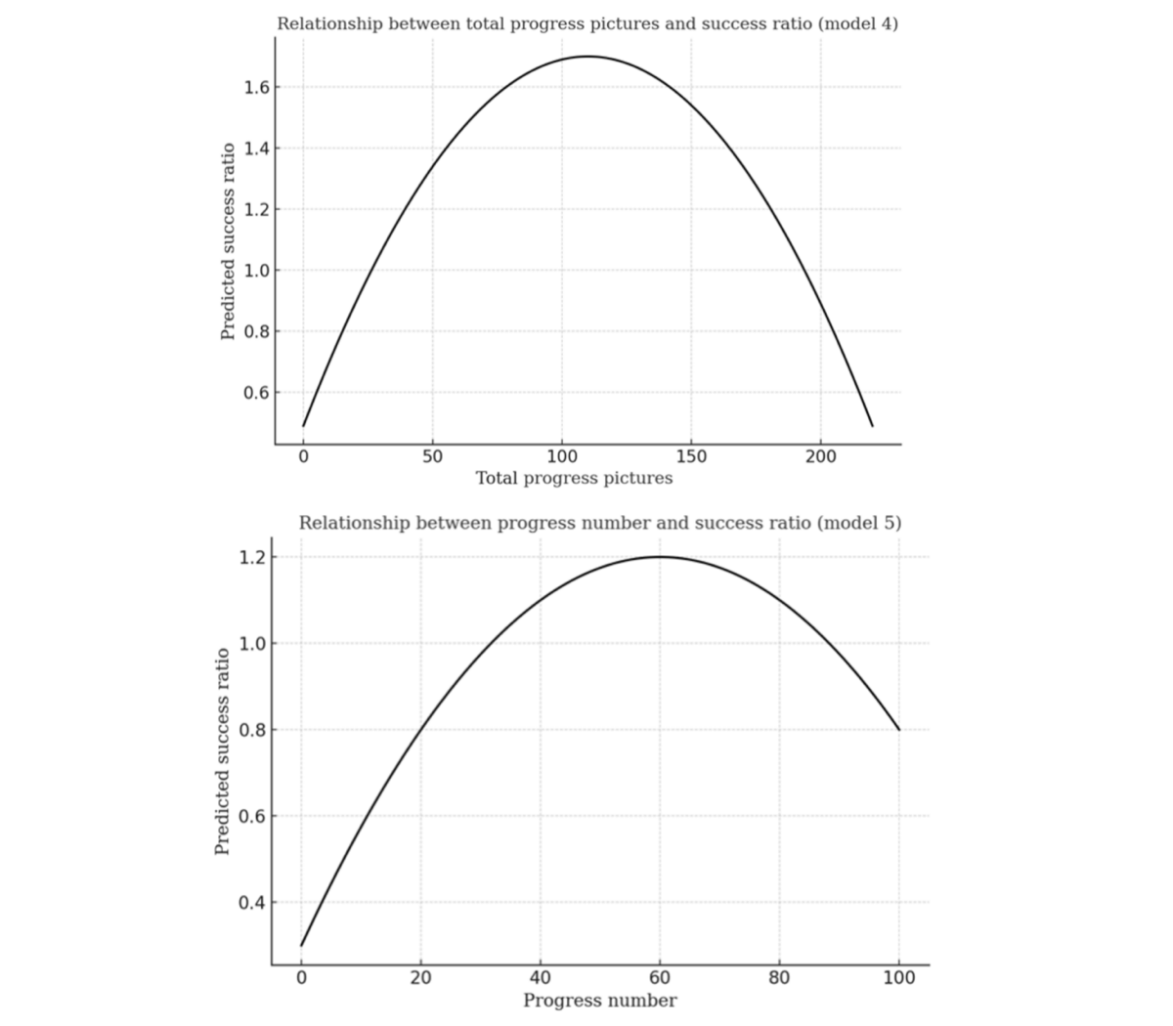
U-test results for models 4 and 5.

**Figure 7:**
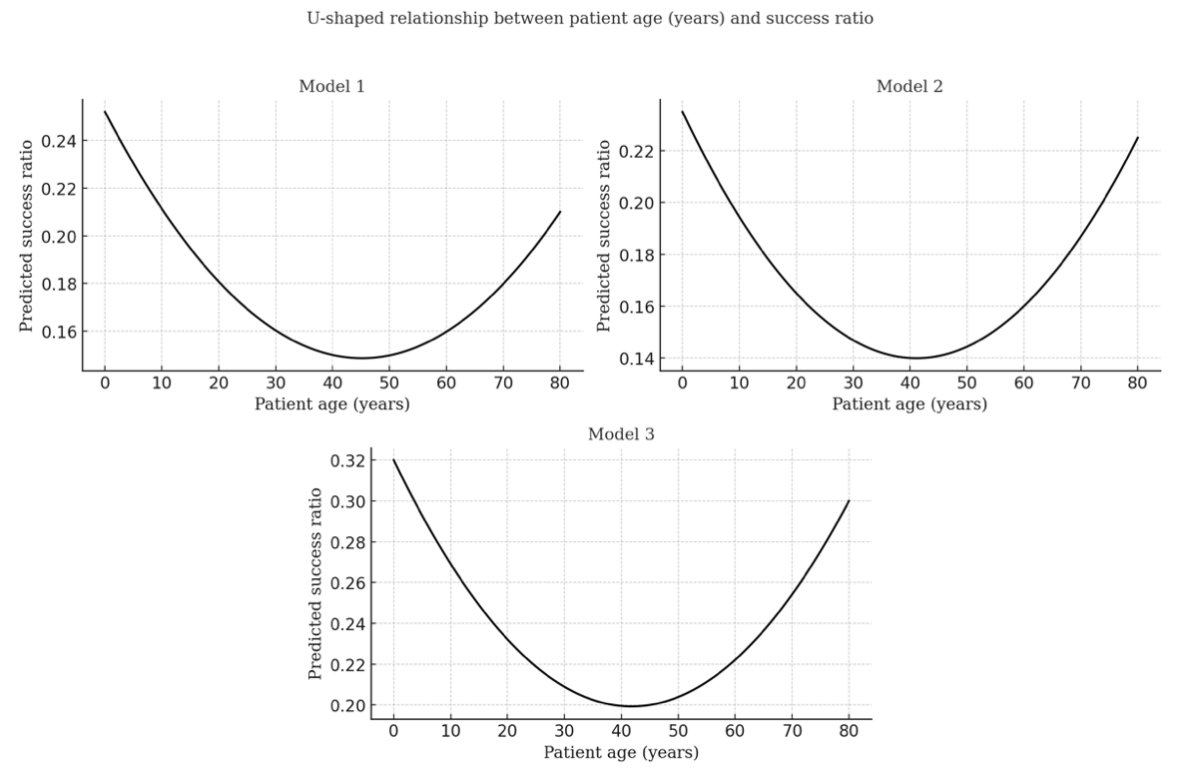
U-test results for age.

Overall, we present 6 models using the success ratio (Tables 3-5) and success indicator (Tables 5 and 6). U-shaped relationships are visualized in Figures 5-7.

**Table 3:** Regression results for success ratio (models 1-3). This table presents regression results for narrative affordance models (models 1-3) with success ratio as the dependent variable. Model (a) shows main effects; model (b) adds U-tests for significant predictors.

	Model 1a		Model 1b			Model 2a			Model 2b			Model 3a			Model 3b	
	T-value	*P* value	T-value	*P* value	T-value		*P* value	T-value		*P* value	T-value		*P* value	T-value		*P* value
Title length	2.112	.04	0.611	.54	—^a^		—	—		—	2.252		.03	0.000		>.99
I(title length)^2	N/A^b^	N/A	–0.448	.65	—		—	—		—	—		—	0.144		.89
Title age	–1.652	.10	–1.818	.07	—		—	—		—	–0.343		.73	–0.342		.73
Title gender	–0.776	.44	–0.734	.46	—		—	—		—	–0.784		.43	–0.730		.47
Title disease	2.583	.01	2.517	.01	—		—	—		—	2.940		.003	2.806		.005
Title negative words	–0.190	.85	–0.297	.77	—		—	—		—	–0.504		.62	–0.382		.70
Title positive words	–1.452	.15	–1.304	.19	—		—	—		—	–1.788		.07	–1.757		.08
Title sentiment	–2.286	.02	0.173	.86	—		—	—		—	0.401		.69	0.623		.53
I(title sentiment)^2	—		–0.624	.53	—		—	—		—	—		—	—		—
Title degree words	1.767	.08	1.723	.09	—		—	—		—	1.422		.16	1.360		.17
Title degree score	1.113	.27	1.071	.28	—		—	—		—	–0.961		.34	–0.860		.39
Story length	—	—	—	—	1.116		.27	1.131		.26	0.319		.75	0.626		.53
Story age	—	—	—	—	0.817		.41	1.273		.20	0.554		.58	0.897		.37
Story gender	—	—	—	—	1.288		.20	1.263		.21	1.764		.08	1.483		.14
Story negative words	—	—	—	—	0.384		.70	0.220		.83	–0.493		.62	–0.473		.64
Story positive words	—	—	—	—	–0.202		.84	–0.044		.97	–0.317		.75	–0.225		.82
Story sentiment	—	—	—	—	—		—	—		—	–0.657		.51	–0.650		.52
Story degree words	—	—	—	—	–0.683		.50	–0.805		.42	1.154		.25	–1.336		.18
Story degree score	—	—	—	—	0.299		.77	0.414		.68	1.139		.26	1.257		.21
Purpose length	—	—	—	—	—		—	—		—	–2.754		.006	0.130		.90
I(purpose length)^2	—	—	—	—	—		—	—		—	—			–0.840		.40
Purpose sentiment	—	—	—	—	—		—	—		—	0.568		.57	1.175		.24
Plan length	—	—	—	—	—		—	—		—	–1.303		.19	–1.626		.10
Plan sentiment	—	—	—	—	—		—	—		—	2.160		.03	3.990		<.001
I(plan sentiment)^2	—	—	—	—	—		—	—		—	—		—	–3.717		<.001
Surplus length	—	—	—	—	—			—		—	2.139		.03	3.335		.001
I(surplus length)^2	—	—	—	—	—			—		—	—		—	–2.838		.005
Surplus sentiment	—	—	—	—	—			—		—	–0.252		.80	–2.153		.03
Patient age (years)	–3.701	<.001	–3.456	.001	–3.119		.002	–3.604		<.001	–2.699		.007	–3.138		.002
I(patient age)^2	—	—	2.420	.02	—		—	2.791		.005	—		—	2.409		.02
Patient gender	–1.499	.13	–1.484	.14	–0.764		.45	–0.796		.43	–1.061		.30	–1.300		.19
Beneficiary indicator	–3.831	<.001	–3.827	<.001	–2.816		.005	–2.770		.006	—		—	—		

^a^Variable was not available and therefore not included in the model.

^b^N/A: not applicable, but included in the model under every table.

**Table 4:** Regression results for success ratio (models 4 and 5). presents regression results for visual and progress affordance models (models 4 and 5) with success ratio as the dependent variable. Model (a) shows main effects; model (b) adds U-tests for significant predictors.

	Model 4a			Model 4b			Model 5a			Model 5b	
	T-value	*P* value	T-value		*P* value	T-value		*P* value	T-value		*P* value
Project description picture number	–1.304	.19	–1.834		.07	—^a^		—	—		—
Progress picture number	9.494	<.001	17.741		<.001	—		—	—		—
I(progress picture number)^2	—	—	–14.734		<.001	—		—	—		—
Progress number	—	—	—		—	6.288		<.001	10.960		<.001
I(progress number)^2	—	—	—		—	—		—	–8.787		<.001
Progress length	—	—	—		—	–0.496		.62	–2.438		.02
Progress sentiment	—	—	—		—	–0.667		.51	–1.049		.29
Patient age (years)	–3.506	<.001	–2.431		.02	–3.138		.002	–2.135		.03
I(patient age)^2	—	—	1.946		.06	—		—	1.536		.13
Patient gender	–1.667	.10	–1.776		.08	–1.921		.06	–1.827		.07
Beneficiary indicator	–0.330	.74	1.857		.06	–1.188		.24	–1.970		.049

^a^Not applicable.

Table 3 presents the regression analyses for the narrativity affordance models (models 1-3), using success ratio as the dependent variable. T-values are reported with *P* values in parentheses. Story sentiment (model 2) and the beneficiary indicator (model 3) are marked as “N/A” to reflect their exclusion from interpretation due to substantial bias found in robustness tests. Model (a) shows the main effects, while model (b) includes U-tests for variables significant in model (a). U-tests (quadratic terms) are only estimated for continuous predictors. For binary variables, quadratic terms are perfectly collinear with the original variable and therefore omitted from the models.

For H1a, our findings reveal that the impact of text length on success varies by text type, providing partial support for H1a. Surplus use length shows a significant inverted U-shaped impact on the success ratio (*P*=.005), but not for other texts (Figure 5). The main effects suggest that longer text generally leads to greater success. Longer titles (model 1a: *P*=.04; model 3a: *P*=.03) and more detailed surplus fund explanations (*P*=.03) improve the success ratio, while longer fund purpose descriptions (*P*=.006) reduce it.

**Table 5:** Regression results for the overall model. This table presents regression analyses for overall model 6. Model (a) shows main effects; model (b) adds U-tests for significant predictors.

	Success ratio						Success indicator				
	Model 6a			Model 6b			Model 6a			Model 6b	
	T-value	*P* value	T-value		*P* value	T-value		*P* value	T-value		*P* value
Narrative length	–0.288	.77	–0.389		.70	1.856		.06	1.851		.06
Narrative demographic	1.695	.09	–1.397		.16	1.233		.22	0.989		.32
Narrative valence word	–0.185	.85	–0.234		.82	–1.919		.06	–2.076		.04
Narrative degree words	0.224	.82	–0.407		.68	–0.022		.98	–0.198		.84
Narrative degree score	0.088	.93	0.780		.44	0.137		.89	0.311		.76
Project description picture number	–1.594	.11	–1.726		.09	–2.104		.04	–0.661		.51
I(project description picture number)	—^a^	—	—		—	—		—	–0.165		.87
Progress picture number	–0.109	.91	9.079		<.001	–2.848		.004	2.063		.04
I(progress picture number^2)	—	—	—		—	—		—	–1.562		.12
Progress number	4.144	<.001	8.046		<.001	4.009		<.001	3.747		<.001
I(progress number^2)	—	—	–13.008		<.001	—		—	–2.244		.03
Progress length	–0.399	.69	–4.026		<.001	–0.435		.66	–1.890		.06
Progress sentiment	–0.551	.58	–0.718		.47	–0.467		.64	–0.454		.65
Patient age (years)	–3.029	.003	–1.902		.06	–0.881		.38	–0.149		.88
I(patient age^2)	—	—	1.470		.14	—		—	—		—
Patient gender	–1.612	.11	–1.331		.18	–0.359		.72	–0.201		.84
Beneficiary indicator	–1.174	.24	0.148		.88	1.019		.31	1.771		.08

^a^Variable was not available and therefore not included in the model.

**Table 6:** Regression results for the success indicator (models 4 and 5). This table presents regression analyses for visual and progress affordance models (models 4 and 5), using the success indicator as the dependent variable. Model (a) shows main effects; model (b) adds U-tests for significant predictors.

	Model 4a		Model 4b		Model 5a			Model 5b	
	Z-value	*P* value	Z-value	*P* value	Z-value	*P* value	Z-value		*P* value
Project description picture number	–0.095	.92	–0.742	.46	—^a^	—	—		—
Progress picture number	2.289	.02	5.415	<.001	—	—	—		—
I(progress picture number)^2	—	—	–3.298	.001	—	—	—		—
Progress number	—	—	—	—	1.981	.048	3.854		<.001
I(progress number)^2	—	—	—	—	—	—	–2.588		.01
Progress length	—	—	—	—	–0.015	.99	–1.096		.27
Progress sentiment	—	—	—	—	–0.177	.86	–0.416		.68
Patient age (years)	–1.839	.07	–0.444	.66	–1.769	.08	–1.084		.28
Patient gender	–0.569	.57	–0.547	.58	–0.521	.60	–0.418		.68
Beneficiary indicator	0.918	.36	1.290	.20	1.246	.21	0.478		.63

^a^Variable was not available and therefore not included in the model.

Including demographic information does not consistently enhance project success; only when the title includes a disease (model 1a: *P*=.01; model 3a: *P*=.003) does it show a significant positive effect, offering partial support for H1b. H1c is largely supported. A more positive-phrased fund use plan (*P*<.001) significantly improves the success ratio, with a U-shaped pattern (*P*<.001) suggesting moderate sentiment is more effective than extremes (Figure 5). In contrast, greater positivity in titles (*P*=.02) reduces the success ratio.

The results support H2, showing that a higher number of progress update images (*P*<.001) is associated with greater success across both outcome measures, though this relationship follows an inverted U-shaped pattern. Progress-related models confirm an inverted U-shaped relationship between update frequency (*P*<.001) and project success, supporting H3a (Figure 6). Although frequent updates enhance both the success ratio and the likelihood of success, the effect diminishes at higher frequencies. H3b is not supported, as progress update sentiment shows no significant effect on project success.

We conducted interaction analyses only when the corresponding main effects were significant to avoid testing interactions without meaningful baseline effects (Tables S7 and S8 in Multimedia Appendix 1). The success ratio, including gender in the title (*P*=.03), positively affects success, but this effect weakens when the patient is male (*P*=.01). The positive effect of story length is reduced when age is mentioned in the title (*P*=.01), and the negative effect of negative words in the story also diminishes under the same condition (*P*=.02). Moreover, positive sentiment in the story description (*P*=.04) amplifies the positive effect of story length and mitigates the negative effect of negative words. Degree words in the story (*P*=.03) also moderate the impact of negative language, reducing its negative effect when more degree words are present, but enhancing it when the intensity of degree words is higher (*P*=.03).

Additionally, for success ratio, interaction analysis for model 4 reveals a marginally significant positive effect of total progress pictures (*P*=.05). This positive effect weakens for older patients (*P*=.045) but strengthens when the project supports multiple beneficiaries compared with a single patient (*P*<.001). Model 5, focusing on progress updates, shows no significant effect on the success ratio but does influence the likelihood of success. Negative sentiment in progress updates (*P*=.01) reduces success likelihood, especially in single-beneficiary projects (*P*=.03) though more updates mitigate this effect (*P*<.001).

Beyond hypothesis testing and interaction analysis, control variables show consistent effects. Projects with older patients (models 1-6) and individual beneficiaries (models 1 and 2) tend to have lower success ratios. Quadratic tests reveal a U-shaped relationship for patient age across affordances (models 1-3), indicating that both younger and older patients see higher success than middle-aged ones (Figure 7).

The final model integrates all affordances into a cohesive framework. When controlling for all factors, the inclusion of demographic information in narrative text marginally improves the success ratio (*P*=.09). A higher number of progress updates (*P*<.001) significantly enhances both the success ratio and the likelihood of success, with an inverted U-shaped pattern indicating diminishing returns at higher frequencies—consistent with earlier models. Patient age remains significant (*P*=.003), with younger patients more likely to succeed. The success indicator further suggests that more project description images are associated with lower success.

#### Additional Analysis

The XGBoost (extreme gradient boosting) models were implemented for both dependent variables to capture complex relationships between predictors. The regression model demonstrated strong predictive performance, with a root mean square error of 0.00083, indicating a close alignment between predicted and actual donation outcomes. The classification model achieved 91% accuracy, effectively distinguishing successful projects from unsuccessful ones.

To interpret the contributions of individual features to success predictions, we conducted SHAP (Shapley additive explanations) analysis for the success ratio. The analysis identified donation frequency and donation amounts as the most influential predictors, with mean absolute SHAP values of 0.287 and 0.025, respectively. This aligns with expectations, as a higher number of contributions and greater total donations naturally increase the likelihood of success. Beyond these factors, patient age emerged as the second most significant predictor (0.084) (Table 7), highlighting a demographic bias where younger patients tend to receive stronger donor support.

**Table 7:** Shapley additive explanations (SHAP) analysis results.

Variable	Mean absolute
Patient age	0.0841
Title gender	0.0180
Title length	0.0092
Patient gender	0.0007
Progress picture numbers	0.0006
All other variables	0

## Discussion

### Primary Findings

Key findings reveal nuanced effects of textual content: longer, informative titles boost success, while the impact of story length varies by context. Moderate detail in surplus fund explanations and explicit demographic cues, particularly disease and gender (female), enhance appeal. This finding directly contradicts the finding of Wang et al [42] regarding the negative impact of female gender on the success of crowdfunding projects. Emotional framing with negative titles and moderately positive fund use plans engages donors effectively. Visual and progress updates predict success through positive inverted U-shaped relationships. Critically, when controlling for all affordances simultaneously, only the frequency of progress updates maintains a robust, inverted U-shaped positive effect. Finally, patient demographics, especially age extremes, significantly improve success, underscoring the importance of multimodal analysis in understanding crowdfunding outcomes.

This study analyzes 1261 health-related crowdfunding projects from Shuidichou to examine how narrativity, visibility, and progress affordances collectively influence project success. After removing models 1 to 3 for the success indicator due to failed robustness tests, we found highly consistent outcomes between the 2 success measures for the visual, progress, and combined affordance models.

Narrativity plays a critical role in crowdfunding outcomes. While prior research suggests an inverted U-shaped relationship between text length and success [14,16,44], our findings show no such pattern for title or story length, instead indicating a more nuanced effect. Specifically, longer titles consistently improve project success, suggesting that donors favor more informative titles. Given their generally short length (max 13 characters), even small increases may have a noticeable impact. The influence of story length appears context-dependent. Its positive effect weakens when age appears in the title, suggesting informational redundancy may lessen effectiveness. Conversely, a more positive tone in the story strengthens the effect, implying that longer narratives are more impactful when framed optimistically. This may be because a positive tone helps sustain engagement in longer texts by reducing emotional fatigue. In emotionally charged contexts like medical crowdfunding, optimistic framing can also convey resilience and hope, making the project appear more credible and worth supporting.

Beyond the title and main description, the length of surplus fund explanations matters—donors appear to value how extra funds will be used. Moderate detail can build trust and encourage donations [63]. Extending prior research, we find that overly long fund purpose descriptions negatively impact success, possibly because donors assume funds will support severe cases or due to reduced patience with lengthy text [64]. Regarding demographic information in textual content, our findings partially align with prior research. Titles mentioning disease positively influence success [44], likely because they clearly communicate the medical issue, drawing attention and encouraging users to click and engage with the project. However, the inclusion of other demographic details showed no significant effect, failing to replicate findings from previous studies [12,13,18,44,46].

The emotional tone of textual content significantly shapes project success [17,18,57]. Consistent with prior research, a negative tone in titles is effective in increasing donations [44], while positive sentiment in fund use plans enhances success. A U-shaped relationship between plan sentiment and success further suggests that moderate emotional tone may be more persuasive than extreme positivity or negativity. This may indicate that overly cheerful language in serious medical funding explanations may come across as insincere or discordant, whereas a moderately positive tone can convey hope and resilience without downplaying the gravity of the situation. Striking this balance may foster empathy and trust, highlighting the importance of context-appropriate emotional framing. Although overall story sentiment was removed from the robustness test due to potential bias, a higher number of negative words still reduces success. This effect is mitigated by age references in titles or the presence of positive and intensifying language in the story, though a stronger degree of intensity slightly amplifies it. Interestingly, while donors may respond to a negatively framed title, they seem less receptive to a high concentration of negative words in the story. This contrast may stem from donors being drawn in by urgency in titles but feeling overwhelmed by negative narratives in the main story.

Although prior research suggests images may have a stronger impact than text [18], we find no such effect for project description images. In contrast, more images in progress updates enhance project success, though their effectiveness declines when used excessively. The relationship also varies by context, with stronger effects in projects for individual beneficiaries and weaker effects when the patient is older. This may suggest that while donors value helping more people, moderate image use is effective when focused on a single recipient, as it may evoke stronger, individually oriented empathy. In contrast, for older patients, donor preferences may favor younger individuals, making additional images less impactful. Similarly, the number of progress updates supports project success, with an inverted U-shaped pattern—while frequent updates generally encourage greater donations [65], excessive updates may reduce their effectiveness. This highlights the importance of both visual and textual progress affordances, though their impact diminishes when overused.

Departing from prior work that isolates individual factors, our study provides a multimodal analysis of how narrativity, visibility, and progress affordances jointly influence crowdfunding success, capturing the complex dynamics of donor behavior. Although a large body of prior work highlights the significant role of narrativity affordances [12-17,20], our findings differ. After accounting for visual and progress-related cues, most narrative effects become insignificant. Only the inclusion of demographic information shows a marginal positive impact. Similarly, we find no significant effect from visual elements. Notably, progress cues remain strong predictors of project success, with an inverted U-shaped relationship observed. This suggests that in the context of similarly structured crowdfunding projects, donors may rely most on progress updates to guide their decisions. Frequent updates likely signal transparency and build trust, enhancing donor motivation, while excessive updates may lead to fatigue or reduced engagement.

The final research question integrates patient demographic characteristics into the discussion. Building on prior research, this study validates and extends the findings of Ba et al [20] and Zhang et al [23], which found that projects featuring younger patients attract more donations and better meet funding goals across affordance types. Similar to Zhang et al [23], we find that older patients also receive higher donations than middle-aged ones. This pattern underscores the preference for supporting beneficiaries at the extremes of the age spectrum. Furthermore, this study builds on Ba et al’s findings [20] by using advanced techniques, including XGBoost and SHAP models, to examine the nuanced role of patient age. These models consistently identify age as a key predictor of success across all 3 affordance dimensions, reinforcing the importance of demographics in crowdfunding outcomes. Additionally, projects supporting individual beneficiaries appear to weaken the influence of narrative and visual features, suggesting these affordances are more effective in projects aiding multiple recipients.

### Practical Implications

Our findings provide practical guidance for patients and platform managers on structuring crowdfunding projects to maximize donations and funding success. First, our multimodal analysis shows that projects featuring younger or older patients and those supporting multiple beneficiaries attract more donations. Therefore, project descriptions should emphasize these demographic factors. For example, a project supporting patients with blood-related diseases shares the story of both a child and a 29-year-old mother. Focusing on the child’s story could attract more donations, so the project might prioritize highlighting children over detailing the mother’s story, thereby maximizing benefits for all beneficiaries.

Then, for projects benefiting individual patients, platform managers might consider grouping similar cases to improve visibility and broaden appeal. For example, a patient aged 53 years with Parkinson disease may struggle individually but could gain enhanced visibility and attract more donors by being grouped with other Parkinson patients, increasing the likelihood of meeting fundraising targets.

Beyond demographic insights, projects should use longer titles that specify the disease and convey urgency in a negative tone, omitting age details. Generic titles like “Partnering with the Very Sick to Get Through Difficult Times” lack specificity and urgency. Instead, platforms could guide projects to include the specific disease, rather than vague terms such as “very sick.” Moreover, replacing optimistic wording, such as “partnering,” with more urgent language can emphasize the severity. For example, titles such as “A Female with Cancer Needs Urgent Help,” which reached its funding goal of 80,154 CNY (US $11,694), or “Boy’s Leukemia relapsed twice, restarting the fight of survival,” which achieved its funding goal with 800,000 CNY (US $114,555). Well-framed titles can more effectively emphasize the urgency of the need and drive greater engagement.

The interaction suggests that the effectiveness of a title depends on demographic factors such as gender and beneficiary type. Titles with gender details generally have a positive impact, but are less effective for male patients or individual beneficiary projects. To compensate, these projects could emphasize visual updates or progress-related details to enhance engagement. Similarly, including patient age in titles weakens the positive effect of narrative length, especially for male patients. To maintain donor interest, projects should omit age details and focus on emotionally compelling or progress-oriented narratives. Platforms should also ensure surplus fund use plans are clear, while avoiding overly lengthy or repetitive explanations. Tailoring strategies to patient characteristics and optimizing key narrative elements can maximize donor response and crowdfunding success. Importantly, active and transparent treatment updates are crucial, but should not be overly frequent. Our findings show that, when all types of information are available, donors rely most on progress updates. Project managers should therefore encourage patients to maintain regular updates while avoiding excess that could dilute donor attention.

Our findings are meaningful and applicable across diverse crowdfunding platforms. Although some might argue that Shuidichou’s results are limited to a Chinese context, we emphasize that the types of information examined (textual narratives, visual elements, and progress updates) are universally present across platforms. Crucially, our analysis demonstrates that when all affordances are considered simultaneously, the impact of narrative elements diminishes, while the strong positive effect of progress updates persists. This result underscores the importance of examining information integratively, as donors typically encounter all these elements together. Unlike previous studies, our findings distinctly show that dynamic elements (eg, update frequency) have a stronger influence on donor decisions compared with static narrative elements, highlighting a vital direction for future crowdfunding research.

Overall, all findings offer practical guidance for both patients and platform managers. In general, patients should openly share details to build trust, while platform managers should be informed about new research related to crowdfunding practices. Our findings highlight the importance of collaboration between these 2 parties in improving the effectiveness of crowdfunding. The strategic application of affordances can help projects optimize donor engagement and increase the probability of success.

### Limitations and Future Research

While this study provides valuable information on the multimodal impact of information affordance on donations, several limitations should be noted. Due to time constraints and limited access to the data of the Shuidichou platform, we could not extract all health-related projects automatically. Instead, project selection required manual link extraction, potentially leading to omissions within the selected timeframe. To mitigate this, multiple research assistants manually extracted links by navigating from the top to the bottom of the platform pages. Nonetheless, we acknowledge the possibility of omitted cases. Additionally, during extraction, we observed that the platform predominantly features severe illnesses such as cancer or trauma from events like fires, while chronic conditions (eg, vision impairment) are rarely represented. This suggests our results may not fully generalize to chronic disease projects.

Although the initial aim was to extract patients’ names and their relationship with the project creators, these details were not accessible through Python-based methods, limiting our analysis of these personal connections. Additionally, we retained model 2 and model 3 despite each containing one variable with moderate bias to preserve the integrity of the simultaneous information environment that donors encounter on crowdfunding platforms. These variables were excluded from interpretation to mitigate potential bias, and an additional sensitivity analysis was conducted to verify the overall stability of the results. Nonetheless, we acknowledge this as a potential limitation.

Beyond these limitations, this study opens several avenues for future research. While our multimodal approach incorporates visual information through numeric counts, deeper analysis of image content—such as facial expressions or the number of people—could provide richer insights. Further investigation is also needed to understand why donors respond to specific cues, such as disease references in titles or gender mentions in descriptions. Survey-based or scenario-driven experiments may help identify psychological mechanisms that mediate these effects. Importantly, we find that progress updates exert the most consistent influence on project success, even after controlling for other affordances. Future research could explore which types of progress updates (eg, medical and emotional) are most effective in engaging donors.

This study highlights the complexity of crowdfunding dynamics, showing that text length and emotional tone can have contrasting effects depending on context. Studying elements in isolation is insufficient. Future work should adopt a holistic approach, examining how donors interact with the overall platform environment to understand what drives donations and how platform design shapes donor behavior.

## Appendix

Multimedia Appendix 1 Supporting data in Supplementary Tables S1-S8.

## Abbreviations

NLP: natural language processing
SHAP: Shapley additive explanations
XGBoost: extreme gradient boosting

## Data Availability

The datasets analyzed during this study are available from the corresponding author on reasonable request.
