# Supplementary material for: Holistic Influence of Multimodal Medical Crowdfunding Affordances on Charitable Crowdfunding Outcome: Systematic Multimodel Analysis Study
Source: JMIR Med Inform. 2025 Sep 16;13:e75563. doi: 10.2196/75563 (PMC12494109; doi:10.2196/75563)
Supplement: Multimedia Appendix 1 [file medinform_v13i1e75563_app1.docx]

**Multimedia Appendix - Tables**

**Table S1** Definitions of Affordance Independent Variables

|  | **Variable** | **Definition** |
| --- | --- | --- |
| Narrativity | title length | Number of characters in project title |
|  | title negative words | Negative sentiment word count in title |
|  | title positive words | Positive sentiment word count in title |
|  | title degree words | Degree-level word count in title |
|  | title degree score | Sum of degree-level word scores in title |
|  | title sentiment | Title sentiment score |
|  | title age | Binary indicator for age mention in title |
|  | title gender | Binary indicator for gender mention in title |
|  | title disease | Binary indicator for disease mention in title |
|  | story length | Number of characters in story description |
|  | story negative words | Negative sentiment word count in story description |
|  | story positive words | Positive sentiment word count in story description |
|  | story sentiment | Story sentiment score |
|  | story degree words | Degree-level word count in project description |
|  | story degree score | Sum of degree-level word scores in project description |
|  | story age | Binary indicator for age mention in project description |
|  | story gender | Binary indicator for gender mention in project description |
|  | purpose length | Number of characters in purpose statement |
|  | purpose sentiment | Project purpose sentiment score |
|  | plan length | Number of characters in project plan |
|  | plan sentiment | Plan description sentiment score |
|  | surplus length | Number of characters of surplus fund use |
|  | surplus sentiment | Surplus fund use sentiment score |
| Visibility | project description picture number | Picture number in project description |
|  | progress picture number | Picture numbers in progress updates |
| Progress | progress number | Total number of progress updates |
|  | progress length | Total characters in progress updates |
|  | progress sentiment | Average sentiment score of all updates |

**Table S2** Descriptive Statistics for Continuous Variables

| **Continuous Variables** | **Min** | **Max** | **Median** | **Mean** | **Std.** |
| --- | --- | --- | --- | --- | --- |
| success ratio | 0 | 1.01 | 0.04 | 0.18 | 0.27 |
| donate number | 0 | 215,514 | 682 | 4,889.77 | 14,623.27 |
| funding goal | 20,000 | 8,000.00 | 300,000 | 436,860.70 | 544,968.74 |
| donation received | 0 | 3,820,279 | 12,274 | 84,315.76 | 250,996.28 |
| title length | 6 | 13 | 12 | 11.39 | 1.65 |
| title negative words | 0 | 2 | 0 | 0.13 | 0.34 |
| title positive words | 0 | 2 | 0 | 0.19 | 0.41 |
| title sentiment | 0.01 | 1 | 0.9 | 0.8 | 0.24 |
| title degree words | 0 | 3 | 0 | 0.03 | 0.2 |
| title degree score | 0 | 2.25 | 0 | 0.02 | 0.14 |
| story length | 128 | 4,079 | 1,037 | 1,131.57 | 534.03 |
| story negative words | 0 | 20 | 4 | 4.36 | 3.06 |
| story positive words | 0 | 28 | 8 | 8.24 | 3.77 |
| story sentiment | 0 | 1 | 1 | 0.9 | 0.04 |
| story degree words | 0 | 31 | 11 | 11.12 | 4.95 |
| story degree score | 0 | 24.25 | 7.75 | 8.03 | 3.78 |
| purpose length | 0 | 816 | 153 | 171.82 | 100.39 |
| purpose sentiment | 0 | 1 | 0.43 | 0.49 | 0.43 |
| plan length | 0 | 830 | 83 | 95.95 | 60.11 |
| plan sentiment | 0 | 1 | 1 | 0.76 | 0.39 |
| surplus length | 0 | 196 | 31 | 28.87 | 30.83 |
| surplus sentiment | 0.01 | 1 | 1 | 0.9 | 0.21 |
| project description picture number | 1 | 26 | 5 | 6.18 | 3.12 |
| progress picture number | 0 | 214 | 1 | 3.12 | 10.11 |
| progress number | 0 | 109 | 2 | 3.68 | 5.9 |
| progress length | 0 | 11,137 | 176 | 287.97 | 493.64 |
| progress sentiment | 0 | 1 | 0.8 | 0.75 | 0.25 |

**Table S3** Descriptive Statistics for Binary Variables

| **Binary variables** | **Frequency** | **Proportion** |
| --- | --- | --- |
| success indicator | 39 | 0.031 |
| project end indicator | 1228 | 0.974 |
| title age | 242 | 0.192 |
| title gender | 446 | 0.354 |
| title disease | 1040 | 0.825 |
| story age | 959 | 0.761 |
| story gender | 778 | 0.617 |
| beneficiary indicator | 1176 | 0.933 |

**Table S4** Regression Results with Sensitivity Analysis for Success Ratio (Model 1-3)

|  | **Model 1a** | **Model 1c** | **Model 2a** | **Model 2c** | **Model 3a** | **Model 3c** |
| --- | --- | --- | --- | --- | --- | --- |
| title length | 2.112  (.04) | 1.350  (.18) |  |  | 2.252  (.03) | 1.468  (.14) |
| title age | -1.652  (.10) | -1.774  (.08) |  |  | -.343  (.73) | -.432  (.67) |
| title gender | -.776  (.44) | -.875  (.38) |  |  | -.784  (.43) | -.917  (.36) |
| title disease | 2.583  (.01) | 1.948  (.05) |  |  | 2.940 (.003) | 2.778  (.006) |
| title negative words | -.190  (.85) | .039  (.97) |  |  | -.504  (.62) | -.268  (.79) |
| title positive words | -1.452  (.15) | -.720  (.47) |  |  | -1.788  (.07) | -1.269  (.21) |
| title sentiment | -2.286  (.02) | -1.439  (.15) |  |  | .401 (.69) | .429  (.67) |
| title degree words | 1.767  (.08) | 2.093  (.04) |  |  | 1.422 (.16) | 1.564  (.12) |
| title degree score | -1.113  (.27) | -1.363  (.17) |  |  | -.961  (.34) | -1.135  (.26) |
| story length |  |  | 1.116  (.27) | .415  (.68) | .319 (.75) | -.073  (.94) |
| story age |  |  | .817  (.41) | .847  (.40) | .554 (.58) | .579  (.56) |
| story gender |  |  | 1.288  (.20) | 1.180  (.24) | 1.764 (.08) | 1.420  (.16) |
| story negative words |  |  | .384  (.70) | -.455  (.65) | -.493  (.62) | -1.142  (.25) |
| story positive words |  |  | -.202  (.84) | .491  (.62) | -.317  (.75) | .248  (.80) |
| story sentiment |  |  | - | - | -.657  (-.51) | -.732  (.47) |
| story degree words |  |  | -.683  (.50) | -.723  (.47) | -.1.154  (.25) | -1.225  (.22) |
| story degree score |  |  | .299  (.77) | .451  (.65) | 1.139 (.26) | 1.278  (.20) |
| purpose length |  |  |  |  | -2.754 (.006) | -2.966  (.003) |
| purpose sentiment |  |  |  |  | .568 (.57) | .580  (.56) |
| plan length |  |  |  |  | -1.303  (.19) | -1.677  (.09) |
| plan sentiment |  |  |  |  | 2.160 (.03) | 1.983  (.048) |
| surplus length |  |  |  |  | 2.139  (.03) | 1.549  (.12) |
| surplus sentiment |  |  |  |  | -.252  (.80) | 1.139  (.26) |
| patient age | -3.701  (<.001) | -3.349  (.001) | -3.119  (.002) | -2.975  (.003) | -2.699  (.007) | -2.780  (.006) |
| patient gender | -1.499  (.13) | -1.440  (.15) | -.764  (.44) | -.788  (.43) | -1.061  (.29) | -1.274  (.20) |
| beneficiary indicator | -3.831  (<.001) | -4.147  (<.001) | -2.816  (.005) | -3.736  (<.001) |  |  |
| Intercept | 4.280  (<.001) | 4.837  (<.001) | 1.678  (.09) | 2.132  (.03) | 1.125  (.26) | 1.420  (.16) |

a. Table 4 presents the regression analyses for the narrativity affordance models (Models 1-3), using success ratio as the dependent variable. b. T-values are reported with p-values in parentheses. c. Story sentiment (Model 2) and the beneficiary indicator (Model 3) are marked as '-' to reflect their exclusion from interpretation due to substantial bias found in robustness tests. d. Model (a) shows the main effects, while Model (c) replicates Model (a) on filtered data, excluding fully successful cases to assess robustness.

**Table S5** Regression Results with Sensitivity Analysis for Success Ratio (Model 4-5)

|  | **Model 4a** | **Model 4c** | **Model 5a** | **Model 5c** |
| --- | --- | --- | --- | --- |
| project description picture number | -1.304  (.19) | -1.414  (.16) |  |  |
| progress picture number | 9.494  (<.001) | 9.259  (<.001) |  |  |
| progress number |  |  | 6.288  (<.001) | 5.508  (<.001) |
| progress length |  |  | -.496  (.62) | -.175  (.86) |
| progress sentiment |  |  | -.667  (.51) | -.732  (.46) |
| patient age | -3.506  (<.001) | -3.070  (.002) | -3.138  (.002) | -2.739  (.006) |
| patient gender | -1.667  (.10) | -1.691  (.09) | -1.921  (.06) | -2.127  (.03) |
| beneficiary indicator | -.330  (.74) | -1.045  (.30) | -1.188  (.24) | -2.011  (.045) |

a. Table 5 presents regression results for visual and progress affordance models (Models 4–5) with success ratio as the dependent variable. b. T-values are reported with p-values in parentheses. c. Model (a) shows the main effects, while Model (c) replicates Model (a) on filtered data, excluding fully successful cases to assess robustness.

**Table S6** Regression Results with Sensitivity Analysis for Success Ratio (Model 6)

|  | **Model 6a** | **Model 6c** |
| --- | --- | --- |
| narrative length | -.288  (.77) | -1.468  (.14) |
| narrative demographic | 1.695  (.09) | 1.343  (.18) |
| narrative valence words | -.185  (.85) | .962  (.34) |
| narrative degree words | .224  (.82) | .382  (.70) |
| narrative degree score | .088  (.93) | -.092  (.93) |
| project description picture number | -1.594  (.11) | -.578  (.56) |
| progress picture number | -.109  (.91) | 1.323  (.19) |
| progress number | 4.144  (<.001) | 2.592  (.01) |
| progress length | -.399  (.69) | -.163  (.87) |
| progress sentiment | -.551  (.58) | -.462  (.64) |
| patient age | -3.029  (.003) | -3.043  (.002) |
| patient gender | -1.612  (.11) | -1.760  (.08) |
| beneficiary indicator | -1.174  (.24) | -1.950  (.05) |

a. Table 6 presents regression results for overall model 6 with success ratio as the dependent variable. b. T-values are reported with p-values in parentheses. c. Model (a) shows the main effects, while Model (c) replicates Model (a) on filtered data, excluding fully successful cases to assess robustness.

**Table S7** Significant Interaction Effects for Success Ratio

|  | **Model 1** | **Model 3** | **Model 4** |
| --- | --- | --- | --- |
| title gender | 2.254  (.02) |  |  |
| title gender:patient gender | -2.493  (.01) |  |  |
| story length |  | 2.358  (.02) |  |
| story negative words |  | -2.030  (.04) |  |
| title age:story length |  | -2.496  (.01) |  |
| title age:story negative words |  | 2.394  (.02) |  |
| story length:story positive words |  | -2.041  (.04) |  |
| story negative words:story positive words |  | 2.038  (.04) |  |
| story negative words  :story degree words |  | 2.167  (.03) |  |
| story negative words :story degree score |  | -2.222  (.03) |  |
| progress picture number |  |  | 1.959  (.05) |
| prog picture number :patient age |  |  | -2.004  (.045) |
| progress picture number:beneficiary indicator |  |  | 14.862  (<.001) |

a. T-values reported; p-values in parentheses. b. No significant predictors in Models 2 and 5.

**Table S8** Interactions Affecting Success Indicator

|  | **Model 5** |
| --- | --- |
| progress sentiment | -2.554  (.01) |
| beneficiary indicator | -.3.487  (.001) |
| progress number  :beneficiary indicator | 4.450  (<.001) |
| progress sentiment:beneficiary indicator | 2.204  (.03) |

T-values reported; p-values in parentheses. No significant predictors in Model 4.
